# Empathic Conservatives and Moralizing Liberals: Political Intergroup Empathy Varies by Political Ideology and Is Explained by Moral Judgment

**DOI:** 10.1177/01461672231198001

**Published:** 2023-09-15

**Authors:** James P. Casey, Eric J. Vanman, Fiona Kate Barlow

**Affiliations:** 1 The University of Queensland, Brisbane, Australia

**Keywords:** political psychology, polarization, empathy, moral judgment, intergroup relations

## Abstract

Empathy has the potential to bridge political divides. Here, we examine barriers to cross-party empathy and explore when and why these differ for liberals and conservatives. In four studies, U.S. and U.K. participants (total *N* = 4,737) read hypothetical scenarios and extended less empathy to suffering political opponents than allies or neutral targets. These effects were strongly shown by liberals but were weaker among conservatives, such that conservatives consistently showed more empathy to liberals than liberals showed to conservatives. This asymmetry was partly explained by liberals’ harsher moral judgments of outgroup members (Studies 1–4) and the fact that liberals saw conservatives as more harmful than conservatives saw liberals (Studies 3 and 4). The asymmetry persisted across changes in the U.S. government and was not explained by perceptions of political power (Studies 3 and 4). Implications and future directions are discussed.

Political polarization has increased in recent decades, particularly in the United States (Abramowitz & Saunders, 2008). This polarization is not simply about divided opinions (opinion polarization), but it also involves increasing animosity and negativity toward political opponents (Iyengar & Westwood, 2015; Warner & Villamil, 2017). High political polarization has been demonstrated in the United Kingdom in the extended conflict around Brexit and in the United States in the partisan and sometimes violent clashes surrounding the 2020 presidential election. So, what is the solution? U.S. President Joe Biden openly acknowledged partisan animosity in his inaugural address, calling for unity and stating: “We can do this if we open our souls instead of hardening our hearts . . . and if we’re willing to stand in the other person’s shoes.” (The White House, 2021). The message was clear: we need empathy—sympathy for and understanding of another person’s concerns—for our political opponents. This may be easier said than done.

In this article, we examine whether liberals and conservatives find it difficult to show empathy for a political opponent, and if so, why. In pursuing these questions, we acknowledge that research on intergroup empathy overlaps with research on intergroup prejudice. Still, there is unique value in studying empathy alone. Empathy research is helpful in prejudice reduction: increases in empathy reduce prejudice (Finlay & Stephan, 2000). Empathy also plays a unique role in predicting positive intergroup relations beyond negative affect (Brown & Hewstone, 2005).

Due to many different definitions in the research literature, “empathy” is now often considered an umbrella term (Coplan & Goldie, 2014). Here we define empathy as *sympathy for and understanding of another person’s suffering, with an aim to reduce that suffering*. People typically have less empathy for a competing outgroup than for their ingroup (see Vanman, 2016, for a review). This intergroup empathy bias is robust (Cikara et al., 2011).

Based on research on intergroup prejudice in politics, we can make some assumptions about facilitators of, and barriers to, intergroup empathy in politics. Negative political relations in the United States appear to be driven by three core judgments: partisans dislike the opposing group, they think the group’s members are dissimilar to themselves, and they judge the other group’s members to be immoral (Finkel et al., 2020). Likewise, both similarity (see Davis, 2018) and liking (Batson et al., 2005) have been found to predict empathy. The link between moral judgment and empathy has not been directly tested, although it is sometimes implied. For example, Batson et al. (2007) found that people had more empathy for targets who showed prosocial behavior than targets who showed antisocial behavior. Although this could be interpreted as a study of moral and immoral behaviors, the authors intended it as a test of how much the participants valued the target’s welfare. Nevertheless, it seems logical that people would be unwilling to feel sympathy for someone they consider immoral, let alone be inclined to reduce that person’s suffering. Therefore, in the studies here, we tested whether partisans lose empathy for their opponents by way of moral judgment, over and above the effects of perceived similarity and dislike of the opponent.

We also investigated whether liberals and conservatives find it equally difficult to empathize with their opponents. The literature on partisan differences is mixed and heavily debated. On one hand, much research appears to suggest that polarization among conservatives has worse outcomes than polarization among liberals (Jost et al., 2020). For example, conservatives were found to be more susceptible to conspiracy beliefs (van der Linden et al., 2021). Accordingly, meta-analyses show that conservatives are generally more sensitive to threat, less tolerant of uncertainty, and less open to experience than liberals (Jost et al., 2003, 2017)—traits that may make them less likely to have empathy for their opponents. On the other hand, these personality differences do not always translate to asymmetries in political behavior. For example, liberals and conservatives were shown to be equally biased in processing political information, even though simultaneously liberals showed more openness to experience (Eichmeier & Stenhouse, 2019). Therefore, we cannot assume that conservatives will be less empathic than liberals based only on differences in personality.

A second point of contention in this literature is whether asymmetries in study design cause the asymmetries in the results. Although liberals show less prejudice than conservatives to specified targets (Sibley & Duckitt, 2008), these specific targets tend to be low-status, disadvantaged, and aligned with politically liberal causes. When considering a range of targets across the political spectrum, liberals and conservatives showed different levels of prejudice according to the target’s alignment with partisan values. Moreover, liberals and conservatives showed equal prejudice toward *each other* (Brandt & Crawford, 2020).

Accordingly, in research on political differences in *empathy*, some studies show that liberals are more empathic than conservatives: liberals rate themselves as higher in empathy than conservatives do (Iyer et al., 2012), and ratings of empathy are correlated with support for liberal policies (Waytz et al., 2016, p. 62; see Morris, 2020, for a review). However, these studies frequently measure trait empathy for nonspecific targets, as in the Interpersonal Reactivity Index (e.g., “I would describe myself as a pretty soft-hearted person”; Davis, 1980). Few studies have compared partisans’ empathy for specific targets (Morris, 2020), let alone for each other. It may be that liberals’ general tendency toward empathy does not extend to disliked outgroups that they consider immoral (i.e., conservatives).

One study has directly examined empathy for one’s political opponents. In Hasson et al. (2018), participants from the United States, Israel, and Germany rated their empathy for political protestors injured during a protest. These protestors either shared the same political ideology as the participant (allies), the opposite ideology (opponents), or their politics were unspecified. Liberal participants were found to have more empathy *in absolute terms* than conservatives for each type of target (allies, opponents, or neutral targets). However, liberals and conservatives reduced their empathy for their opponents by the same amount *relative to empathy for their allies*. From this research, one might conclude that liberals find it easier than conservatives to extend empathy to their opponents, or equally as difficult, depending on how that difference in empathy is measured.

To summarize, in all studies, we tested whether the political intergroup empathy bias was shown—and shown equally—by liberals and conservatives. We tested whether moral judgment explained the political intergroup empathy bias and political asymmetries in this bias over and above the effects of liking and similarity (Studies 1 and 2). We continued to test the role of moral judgment in explaining these asymmetries in Studies 3 to 4 but extended this question to explore possible precursors to moral judgment of political opponents, and whether political power might be responsible for asymmetries in the empathy bias.

## Study 1

The initial study was conducted using a U.S. sample as much of the research on political polarization has occurred in the United States (Abramowitz & Saunders, 2008; Iyengar & Westwood, 2015; Warner & Villamil, 2017).

## Method

Full details of all studies in this article, including preregistrations, all measures, and data sets, are available at https://osf.io/xyr92/. All of the studies in this article were preregistered (Study 1: https://osf.io/q8ms7; Study 2: https://osf.io/4rpx7; Study 3: https://osf.io/9xz85; Study 4: https://osf.io/34tkm) and all preregistrations included the study design, a pre-planned stopping rule, inclusion/exclusion criteria, and planned analyses. We report all preregistered analyses either in the main body of the article or in the Supplementary Materials, and we indicate all deviations from the preregistered methods in the Supplementary Materials. The Ethics Committee at the University of Queensland approved all studies in this article.

### Participants

In March 2020, we recruited U.S. participants through Prolific. The final sample included 549 participants: 270 conservatives and 279 liberals, *M*_age_ = 37.19, *SD* = 12.84; 276 female (50%), 267 male (49%), six gender diverse (1%); 441 White/Caucasian (80%), 24 Asian/Asian American (4%), 20 Black/African American (4%), 64 of widely varying other ethnicities (12%). Participants were each paid £1.05. Details of power analyses and exclusions for each of the four studies in this article are provided in the Supplementary Materials (p. 7-22).

### Design

The study used a between-groups factorial design with the measured variable of participant politics (two levels: liberal or conservative) and the manipulated variable of relative group membership (three levels: ingroup, outgroup, or neutral).

### Procedure

Participants read information about the study and provided consent. The rest of the survey took place in two sections: the experimental manipulation (including the empathy measures and other direct responses to the stimuli) and the demographics (including measures of participants’ politics). The order of these sections was counter-balanced between participants. Participants were fully debriefed at the end of the study.

### Stimuli

Participants read a description of a target person, a 30-year-old American man or woman (randomly selected) who was employed as an assistant manager in a retail store and married with a young child. To manipulate target group membership, this person was described as either conservative and Republican, liberal and Democrat, or no political information was provided. Next, the participant read one of four scenarios describing the target person suffering in some way (e.g., having a sprained ankle; full stimuli in SOM, p. 4).

### Measures

#### Predictor Variables

##### Participant Politics

Participants explicitly identified as either conservative or liberal on their Prolific profile.

#### Outcome Variables

##### Empathic Concern

This measure used three items from Batson et al. (2005): *sympathetic, warm*, and *compassionate.* Participants were asked, “How do you feel about this person?” and responded on a sliding scale (0 = *Not at All*, 10 = *A Great Deal*). Items were averaged (α = 0.89).

##### Perspective-Taking

This measure used two novel items based on trait measures of perspective-taking (e.g., Davis, 1980): *I can imagine how this person would be feeling* and *I can understand what this person would be going through*. Participants were asked, “How much do you agree with these statements?” (−5 = *Strongly Disagree, 0 = Neither Agree nor Disagree* and 5 = *Strongly Agree*). These items were averaged (*r* = .82).

##### Empathic Intentions

This measure used two novel items: *I would try to cheer this person up if I met them* and *I would do my best to help this person if they asked me to* (−5 = *Strongly Disagree* to 5 = *Strongly Agree*). These items were averaged (*r* = .66).

##### Empathic Avoidance

This measure used two novel items: *I would avoid interacting with this person* and *This person deserved what happened to them* (−5 = *Strongly Disagree* to 5 = *Strongly Agree*). These items were averaged (*r* = .48).

Note that the exact breakdown of the empathy items was determined by factor analysis in Study 1 (see SOM, p. 20 for details).

#### Mediators

We included measures of three potential mediators; these items were presented together, but their order was randomized. Participants were asked, “How much do you agree with these statements?” (−5 = *Strongly Disagree*, 0 = *Neither Agree nor Disagree* and 5 = *Strongly Agree*).

##### Similarity

This measure used one item based on Batson et al. (2005): *This person seems to be similar to me*.

##### Liking

This measure used one item: *I think I would like this person if I met them*.

##### Morality

This measure used two items: *This person is probably a good person* and *This person is likely to have poor moral values* (reverse-scored). These items were averaged (*r* = .64).

## Results

For additional statistical detail (e.g., all pairwise comparisons and calculations of *p*-value thresholds), refer to the Supplementary Materials (p. 26).

### The Intergroup Empathy Bias

A two-way multivariate analysis of variance (MANOVA) revealed a main effect of relative group membership on the four measures of empathy, *F*(8, 1,080) = 5.02, *p* < .001, Λ = .930, as well as a main effect of participant politics, *F*(4, 540) = 2.44, *p* = .046, Λ = .982. These main effects were qualified by a Relative Group Membership × Participant Politics interaction, *F*(8, 1,080) = 3.22, *p* = .001, Λ = .954.

We followed up these multivariate effects with a series of univariate analyses of variance (ANOVAs), using pairwise comparisons to follow up the univariate omnibus effects. We used a corrected threshold for significance of *p* = .015 for each test to reduce the risk of Type I error (Derringer, 2018) and further corrected for familywise error when conducting multiple comparisons using the Holm method (Holm, 1979).

Univariate tests showed main effects of relative group membership on empathic concern, intentions, and avoidance but not on perspective-taking. The pattern was the same for each outcome variable: participants showed less empathic concern, less empathic intentions, and more avoidance of outgroup targets than either ingroup or neutral targets.

The univariate main effect of participant politics was significant at *p* < .015 for empathic avoidance only. The trend across all measures was for conservatives to show greater empathy than liberals.

**Figure 1.**
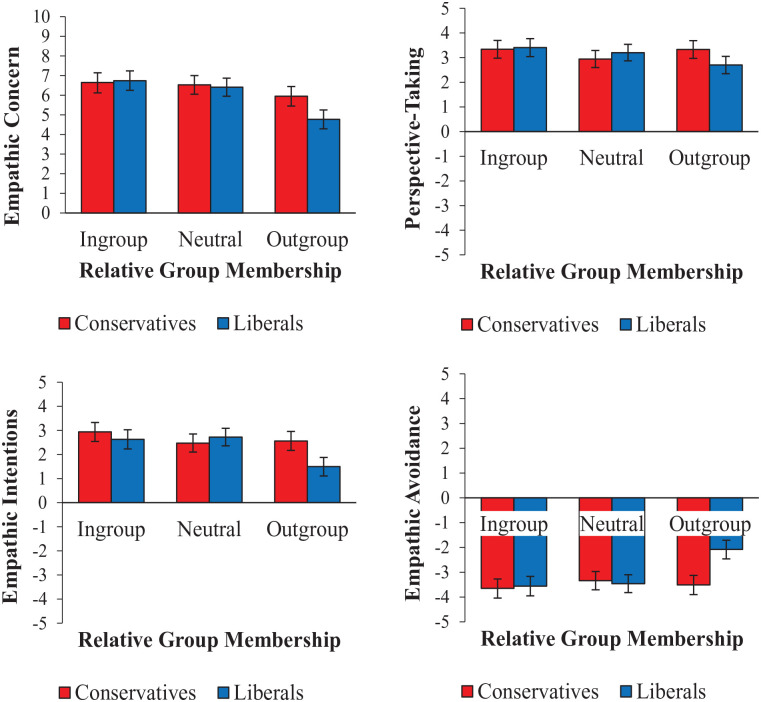
Mean Strength of Empathic Concern (Top Left) and Agreement With Perspective-Taking (Top Right), Empathic Intentions (Lower Left), and Empathic Avoidance (Lower Right) for Ingroup, Outgroup, and Neutral Targets, as Rated by Conservative and Liberal Participants. *Note.* Error bars indicate 95% confidence intervals.

There were significant univariate Relative Group Membership × Participant Politics interactions for empathic intentions and avoidance (at criterion *p* < .015) and non-significant interactions for empathic concern and perspective-taking (*p* = .024 and .031, respectively). Given the multivariate interaction, we ran follow-up tests on all four outcome variables; the interaction terms and cell means for each outcome are shown in Table 1, and the pattern of results is visualized in Figure 1. The pattern was the same for empathic concern, intentions, and avoidance: liberals who saw outgroup targets showed reduced empathy compared with liberals who saw either ingroup or neutral targets, whereas conservatives showed no differences in empathy across the three target conditions. In comparisons across participant politics, liberals showed less empathy for outgroup targets (conservatives) than conservatives showed for outgroup targets (liberals). No pairwise comparisons were significant for ratings of perspective-taking.

**Table 1.** Omnibus Effects and Cell Means for Interaction of Relative Group Membership and Participant Politics on Empathy Variables.

Variable	ω^2^	*df*	*df* error	*F*	*p*	Group condition	Means	
							Conservatives	Liberals
Empathic concern	0.01	2	543	3.74	.024	Ingroup	6.65_a_	6.74_a_
						Neutral	6.53_a_	6.41_a_
						Outgroup	5.95_a_	4.77_b_
Perspective-taking	0.01	2	543	3.51	.031	Ingroup	3.34_a_	3.41_a_
						Neutral	2.94_a_	3.20_a_
						Outgroup	3.33_a_	2.70_a_
Empathic intentions	0.02	2	543	5.77	.003*	Ingroup	2.94_a_	2.63_a_
						Neutral	2.47_a_	2.72_a_
						Outgroup	2.56_a_	1.50_b_
Empathic avoidance	0.03	2	543	9.60	<.001*	Ingroup	−3.65_a_	−3.56_a_
						Neutral	−3.34_a_	−3.46_a_
						Outgroup	−3.51_a_	−2.08_b_

*Note.* For any given outcome variable, differing subscript within a single row or column indicates where conditions differed, *p* < .015, after Holm corrections.

* *p* < .015.

### Moderated Mediation

**Figure 2.**
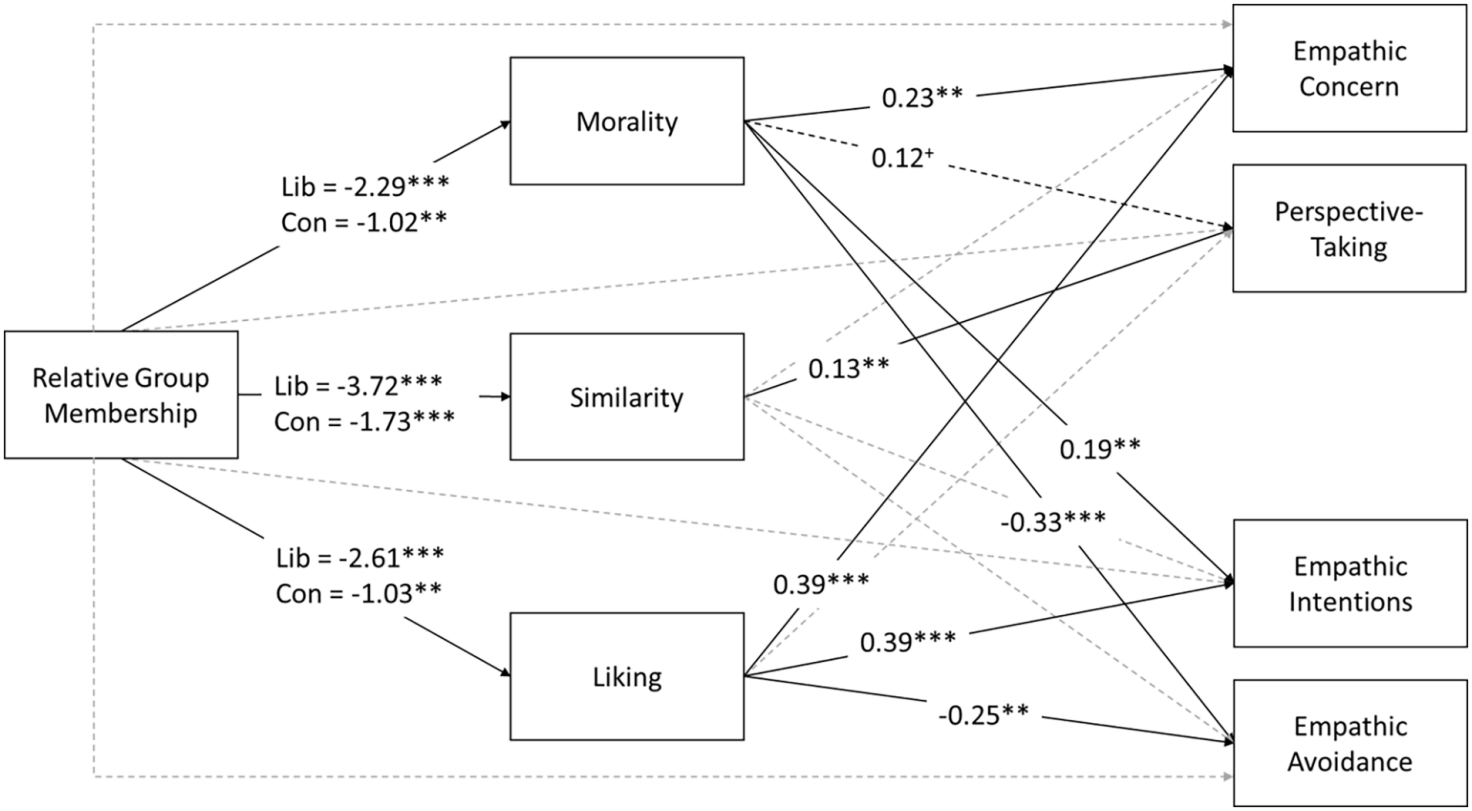
Path Diagram Showing Effects (Unstandardized) of Relative Group Membership on Empathy by Way of Perceptions of Morality, Similarity, and Liking, Moderated by Participant Politics. *Note*. Parallel mediation analyses were conducted on each empathy variable separately, but we have visualized these analyses as a single path diagram for comparison. Relative group membership was coded as outgroup = 1, ingroup = 0. Gray dashed lines indicate paths that were tested but were non-significant. **p < .01. ***p < .001. ^+^*p* < .10.

To explain differences in empathy for ingroup and outgroup targets, we conducted a series of moderated, parallel mediation analyses, excluding data from the neutral condition. The models for these analyses are shown in Figure 2. Analyses showed that relative group membership was indirectly associated with empathy for the target person through its effects on perceived morality and liking of the target. However, these paths were moderated by the politics of the participant (indirect effects and moderation indices are provided in Table 2). Liberal participants rated conservatives as less moral and likable compared with conservatives’ ratings of liberals. These lower ratings of morality and liking statistically explained the pattern whereby liberals reported less empathic concern and intentions and more avoidance of conservative targets, than did conservatives for liberal targets. Conservatives who saw an outgroup target (a liberal) also judged them to be less moral and less likable than ingroup targets, but the difference was smaller than for liberals rating conservatives. In contrast to morality and liking, ratings of target similarity only mediated the effect of relative group membership on perspective-taking. However, as the overall effect of relative group membership on perspective-taking was non-significant in the ANOVA, the mediation by target similarity does not appear to be meaningful.

**Table 2:** Mediated Effects of Relative Group Membership on Empathic Concern, Perspective-Taking, Empathic Intentions, and Empathic Avoidance, Moderated by Participant Politics

Mediator	W (participant politics)	Indirect effect (ab)	95% confidence interval		Index of moderated mediation	95% confidence interval	
			Lower	Upper		Lower	Upper
Empathic concern							
Morality	Liberal	−0.54	−0.945	−0.173	0.30	0.063	0.616
	Conservative	−0.24	−0.484	−0.059			
Liking	Liberal	−1.02	−1.580	−0.525	0.62	0.234	1.110
	Conservative	−0.40	−0.743	−0.141			
Similarity	Liberal	−0.17	−0.626	0.279	0.09	−0.163	0.346
	Conservative	−0.08	−0.334	0.127			
Perspective-taking							
Morality	Liberal	−0.26	−0.610	0.044	0.15	−0.023	0.392
	Conservative	−0.12	−0.298	0.022			
Liking	Liberal	−0.09	−0.445	0.260	0.05	−0.165	0.286
	Conservative	−0.04	−0.194	0.101			
Similarity	Liberal	−0.49	−0.913	−0.123	0.26	0.054	0.565
	Conservative	−0.23	−0.452	−0.047			
Empathic intentions							
Morality	Liberal	−0.43	−0.787	−0.095	0.24	0.037	0.526
	Conservative	−0.19	−0.390	−0.034			
Liking	Liberal	−1.03	−1.481	−0.633	0.62	0.277	1.034
	Conservative	−0.41	−0.725	−0.146			
Similarity	Liberal	0.16	−0.200	0.525	−0.09	−0.309	0.104
	Conservative	0.08	−0.098	0.257			
Empathic avoidance							
Morality	Liberal	0.76	0.360	1.230	−0.42	−0.828	−0.120
	Conservative	0.34	0.118	0.624			
Liking	Liberal	0.65	0.178	1.181	−0.40	−0.804	−0.086
	Conservative	0.26	0.050	0.554			
Similarity	Liberal	0.05	−0.269	0.355	−0.02	−0.199	0.148
	Conservative	0.02	−0.128	0.180			

## Discussion

We found that partisans had less empathy toward their political outgroups than ingroups in a study where the targets were not in a political context and their sufferings were unrelated to their political activity (in contrast to Hasson et al., 2018). Surprisingly, these results were driven by strong effects from liberals. The extent to which conservatives afforded empathy to those suffering was largely unrelated to their political group membership. The finding of increased empathy displayed by conservatives seems to contradict previous research on both sides of the political asymmetry debate (e.g., Brandt & Crawford, 2020; Hasson et al., 2018). However, although we had the statistical power to detect the effect of relative group membership, we did not have sufficient power to reliably detect differences in that effect between liberals and conservatives (see SOM, p. 8 for post hoc analyses), so we cannot yet claim a strong challenge to past research. We addressed this limitation in Study 2.

Our findings are also consistent with the idea that the political empathy bias is explained by moral judgment. When someone feels low empathy for their political opponent, it is not simply because they dislike the other. It is because they think the other is immoral and thus (perhaps) unworthy of empathy (as in the work of Batson et al., 2007). These findings are in line with previous research suggesting partisan prejudice is driven by moral judgment as well as dislike and dissimilarity (Finkel et al., 2020), although in our research, dissimilarity did not play a role once moral judgment and liking were taken into account. These processes may also explain why liberals showed less empathy to outgroup members than conservative participants: liberals judged conservatives more harshly than conservatives did liberals, seeing them as more immoral and less likable.

## Study 2

Study 1 showed a strong empathy bias among liberals yet only a slight empathy bias among conservatives. This finding contradicts previous research (Hasson et al., 2018). It is also surprising given the open hostility seen on both sides of U.S. politics among career politicians and lay citizens (e.g., on social media). In Study 2, we recruited more participants to improve statistical confidence in our results and repeated the study design in a different country (the United Kingdom) to determine whether our findings from Study 1 were reliable and generalizable. We chose the United Kingdom as it is similar politically to the United States (both two-party democratic systems, strongly politically polarized).

## Method

The design, measures, and procedure used in Study 2 were essentially the same as in Study 1, but with phrasing adapted to refer to U.K. targets and political parties. Any other exceptions are noted below.

### Participants

Participants were recruited from Prolific in September 2020. The final sample included 958 participants: 455 conservatives and 503 liberals, *M*_age_ = 37.94, *SD* = 14.74; 594 female (62%), 361 male (38%), three gender diverse (< 1%); 738 Caucasian/White British (77%), 109 simply “British” (11%), 111 of widely varying other ethnicities (12%). Participants were each paid £0.77.

### Stimuli

The stimuli in each condition were identical to those used in Study 1, except the target was now described as British and a supporter of the Conservative Party in the conservative target condition or the Labour Party in the liberal target condition.

### Measures

#### Predictor Variables

##### Participant Politics

Participants identified as either politically Right or Left on their Prolific profile. For consistency, we refer to these groups as conservative and liberal in the rest of this article (for evidence that these labels are appropriate, see SOM, p. 93).

#### Outcome Variables

We ran a factor analysis on the empathy items to determine the factors in play (see SOM, p. 22). This analysis confirmed the same factors of empathic concern and perspective-taking as used in Study 1 (α = .87 and *r* = .75, respectively). However, it failed to produce distinct factors for empathic intentions and avoidance. Instead, these items loaded together on a single factor (α = 0.78; avoidance items reverse-scored). We continue to call this new factor “Empathic Intentions.”

#### Mediators

We included the same three potential mediators as in Study 1: similarity, liking, and morality (*r* = .54).

## Results

For additional statistical detail (e.g., all pairwise comparisons), refer to SOM (p. 39).

### The Intergroup Empathy Bias

A 2 × 3 MANOVA revealed a main effect of relative group membership on the three measures of empathy, *F*(6, 1,898) = 23.14, *p* < .001, Λ = .868, a main effect of participant politics, *F*(3, 949) = 3.75, *p* = .011, Λ = .988, and a Relative Group Membership × Participant Politics interaction, *F*(6, 1,898) = 5.45, *p* < .001, Λ = .966. For follow-up tests, we used a corrected threshold for significance of *p* = .02 (Derringer, 2018) and corrected for familywise error using Holm’s (1979) method.

Univariate ANOVAs showed a consistent main effect of relative group membership. Participants who saw outgroup targets reported less empathic concern, perspective-taking, and empathic intentions than participants who saw ingroup or neutral targets. Participants who saw ingroup or neutral targets did not differ in their ratings.

The univariate main effect of participant politics was found for empathic intentions only: conservatives showed more empathy toward targets than liberals.

**Figure 3.**
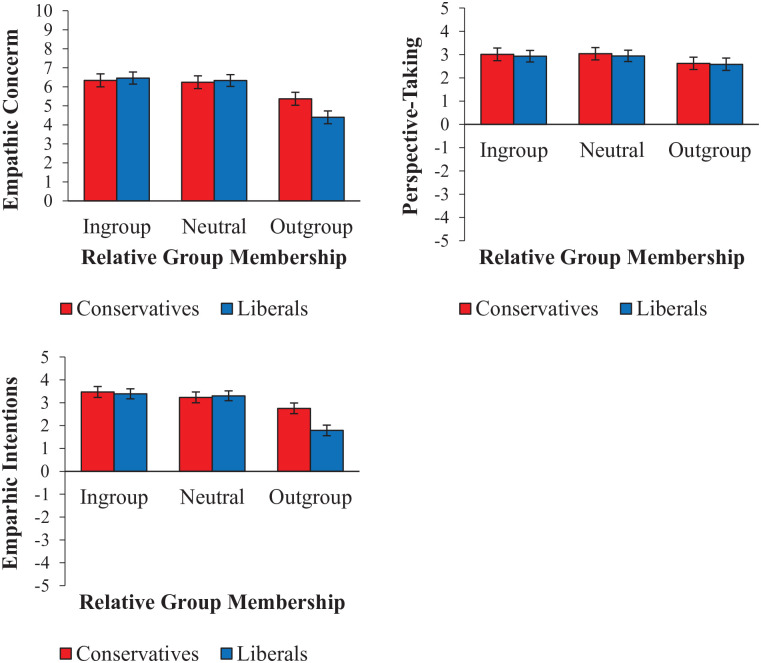
Mean Strength of Empathic Concern (Top Left) and Agreement With Perspective-Taking (Top Right), and Empathic Intentions (Lower Left), for Ingroup, Neutral, or Outgroup Political Targets, as Rated by Conservative and Liberal Participants. *Note.* Error bars indicate 95% confidence intervals.

As shown in Table 3, there were significant univariate Relative Group Membership × Participant Politics interactions for empathic concern and empathic intentions, but not for perspective-taking. Given the multivariate interaction, we ran follow-up tests for all three measures. Follow-up comparisons revealed a similar pattern of effects for empathic concern and intentions: both conservative and liberal participants showed less empathy for outgroup targets compared with ingroup targets (and reduced empathic concern for outgroup targets compared with neutral targets), but the effects were smaller for conservative participants than for liberals. In comparisons across participant politics, conservative participants showed more empathy than liberal participants did for outgroup targets. These effects are visualized in Figure 3.

**Table 3:** Summary of Omnibus Interaction Effects of Relative Group Membership and Participant Politics on Empathy Variables.

Variable	ω^2^	*df*	*df* error	*F*	*p*	Group condition	Means	
							Conservatives	Liberals
Empathic concern	0.01	2	952	6.69	.001*	Ingroup	6.34_a_	6.46_a_
						Neutral	6.24_a_	6.33_a_
						Outgroup	5.37_b_	4.40_c_
Perspective-taking	< 0.01	2	951	0.02	.979	Ingroup	3.01_a_	2.93_a_
						Neutral	3.04_a_	2.94_a_
						Outgroup	2.62_a_	2.58_a_
Empathic intentions	0.02	2	951	11.12	<.001*	Ingroup	3.47_a_	3.39_a_
						Neutral	3.23_a,b_	3.30_a_
						Outgroup	2.75_b_	1.79_c_

*Note.* For any given outcome variable, differing subscript within a single row or column indicates where conditions differed, *p* < .02, after Holm corrections.

* *p* < .02.

### Moderated Mediation

**Figure 4.**
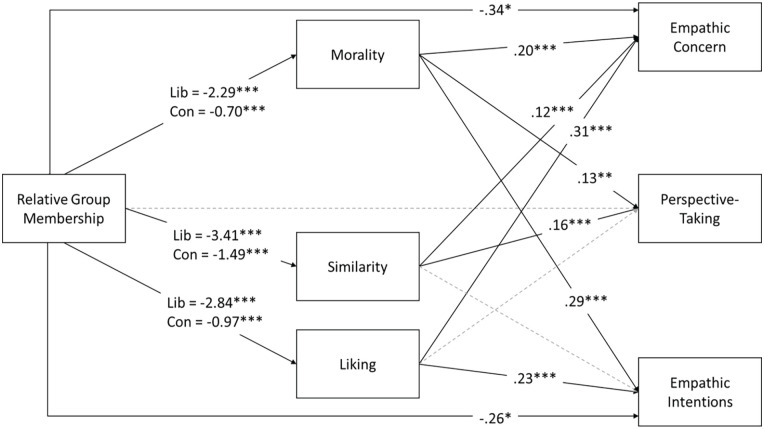
Path Diagram Showing Effects (Unstandardized) of Relative Group Membership on Empathic Concern, Perspective-Taking, and Empathic Intentions by Way of Perceptions of Morality, Similarity, and Liking, Moderated by Participant Politics. *Note*. Parallel mediation analyses were conducted on each empathy variable separately, but we have visualized these analyses as a single path diagram for comparison. Relative group membership was coded as outgroup = 1, ingroup = 0. Gray dashed lines indicate paths that were tested but were non-significant. *p < .05. ***p < .001.

To explain differences in empathy for ingroup and outgroup targets, we again conducted a series of moderated, parallel mediation analyses. The analysis models are visualized in Figure 4, and model coefficients are provided in Table 4. As in Study 1, analyses revealed that relative group membership was indirectly associated with empathy through perceived morality and liking of the target (but also through perceived similarity, unlike in Study 1), and these paths were moderated by the politics of the participant. Liberal participants who saw an outgroup target rated them as less moral, less similar to themselves, and liked them less than liberal participants who saw ingroup members; in turn, these judgments were associated with less empathic concern, less perspective-taking, and less empathic intentions. Conservative participants also showed these patterns but less than half as strongly as the liberal participants.

**Table 4.** Mediated Effects of Relative Group Membership on Empathic Concern, Perspective-Taking, and Empathic Intentions, Moderated by Participant Politics.

Mediator	W (participant politics)	Indirect effect (ab)	95% confidence interval		Index of moderated mediation	95% confidence interval	
			Lower	Upper		Lower	Upper
Empathic concern							
Morality	Liberal	−0.46	−0.742	−0.197	0.31	0.122	0.549
	Conservative	−0.14	−0.261	−0.052			
Liking	Liberal	−0.79	−1.171	−0.459	0.51	0.272	0.819
	Conservative	−0.27	−0.468	−0.126			
Similarity	Liberal	−0.40	−0.675	−0.149	0.22	0.77	0.405
	Conservative	−0.17	−0.327	−0.058			
Perspective-taking							
Morality	Liberal	−0.30	−0.538	0.059	0.21	−0.039	0.395
	Conservative	−0.09	−0.193	0.015			
Liking	Liberal	−0.06	−0.335	0.222	0.04	−0.150	0.221
	Conservative	−0.02	−0.123	0.077			
Similarity	Liberal	−0.55	−0.801	−0.332	0.31	0.153	0.499
	Conservative	−0.24	−0.404	−0.117			
Empathic intentions							
Morality	Liberal	−0.65	−0.909	−0.438	0.45	0.260	0.682
	Conservative	−0.20	−0.336	−0.089			
Liking	Liberal	−0.64	−0.920	−0.389	0.42	0.232	0.647
	Conservative	−0.22	−0.364	−0.102			
Similarity	Liberal	−0.05	−0.215	0.111	0.03	−0.063	0.130
	Conservative	−0.02	−0.099	0.049			

## Discussion

These findings demonstrate a clear political intergroup empathy bias and the first known demonstration of this bias in a U.K. sample. The reduction in empathy was explained by participants’ judgments of outgroup targets as less moral, less likable, and less similar to the self than ingroup targets. Again, we found that the politics of the participant moderated these effects: liberals had more severe judgments of conservatives’ morality, likability, and similarity to themselves and through these variables reported less empathy for conservatives than conservatives did for liberals.

With improved statistical power, we can be confident in the asymmetry between liberals and conservatives. This asymmetry is in the opposite direction to some previous findings where conservatives were generally less empathic than liberals (Hasson et al., 2018; Morris, 2020). The asymmetry is also surprising if conservatives are less tolerant of ambiguity and more sensitive to threat than liberals (Jost et al., 2003, 2017). However, our results do fit with emerging research that challenges some assumed differences between conservatives and liberals. For example, conservatives were better at representing liberals’ beliefs (perspective-taking) than liberals were at representing conservatives’ beliefs (Graham et al., 2012). Similarly, some recent studies have found that conservatives are *more* tolerant of ambiguity than liberals (Sargent & Newman, 2021) and *less* sensitive to some forms of threat (Conway et al., 2021; Eadeh & Chang, 2020), both of which may be related to their capacity for intergroup empathy.

How do we reconcile our results with previous research? In a recent review, Jost et al. (2020) acknowledge that the asymmetry found in their research is not immutable: contextual features can dampen or even reverse this asymmetry, and identifying these features represents progress in the study of political ideology. At certain times or in certain contexts, the intergroup empathy bias will be stronger or weaker for liberals or conservatives. The task is to identify which feature of our research caused this change—what could make conservatives unusually kind to their opponents yet leave liberals unaffected?

One prominent possibility is political power. Studies 1 and 2 took place in countries led at the time by arguably populist conservative leaders (Donald Trump in the United States and Boris Johnson in the United Kingdom), which seems likely to have affected partisans’ opinions of each other. Groups with higher status downplay conflicts between themselves and lower-status groups (Livingstone et al., 2021), ostensibly to maintain and legitimize their position of power. Thus, conservatives’ higher power in Studies 1 and 2 might have motivated them to downplay their conflicts with liberals, allowing them to extend more empathy toward liberals than liberals did to conservatives. We tested this explanation in Study 3.

## Study 3

To account for differences in political power as an explanation for political asymmetries in moral judgment and empathy, we needed to test political intergroup empathy in contexts where liberals had more political power than conservatives. Fortunately for our research, the 2020 U.S. presidential election shifted the balance of power from conservative (Republican) to liberal (Democrat), so we had a unique opportunity to examine this question in otherwise similar conditions to Study 1.

We had competing predictions about whether we would still find differences between liberals and conservatives in the intergroup empathy bias under the new liberal administration. Perhaps our findings would flip completely so liberals would be more empathic (and less morally judgmental) to conservatives than conservatives were to liberals. However, we doubted the changes would be so clean given that a change of government is not a clean experimental manipulation; we could not account for all the contextual elements that would change between the two time-points. To prepare for the possible variations in our results, we also measured participants’ perceptions of their group’s power. This way, we could test whether people who *felt* their party was more powerful had more empathy for their political opponents than people who felt their party was less powerful. Even if we found that liberals and conservatives had equal empathy biases, we could still address when and why these biases differed.

We hypothesized that ingroup power would moderate the size of the empathy gap: that is, liberals and conservatives who perceived their ingroup to have more power would have smaller empathy biases, in line with research on intergroup status (Livingstone et al., 2021). We also hypothesized that any differences between liberals and conservatives in the size of the empathy bias would be because of perceived power. Whichever group had higher perceived power should also have the smaller empathy bias.

We continued to examine moral judgment as a theoretical precursor to reduced empathy. If political power influences the size of the empathy bias, it should do so because it first influences the bias in moral judgment. In this study we did not compare the effect of moral judgment to liking and similarity as in Studies 1 and 2. Instead, we sought to extend previous findings by testing for theoretical precursors to moral judgment. Upon discovering that a target person is a political opponent or political ally, what other assumptions would a partisan make that lead to moral judgments of the target? We identified two likely contenders: *shared identity* with the target (or lack thereof) and perceptions of harm caused by the target’s group (*group harm*).

The measure of shared identity grounds our work more explicitly in the social identity perspective (Turner & Reynolds, 2003). Following this perspective, judgments of others should be more favorable if someone primarily categorizes themselves and the other as part of the same group (such as “American” or “human”) rather than part of distinct and separate groups (Republican versus Democrat).

The measure of group harm is relevant because of the transference of attitudes from the group to the individual. We expect that when a partisan judges an opponent harshly, it is not because they know the other has personally acted in an immoral way; it is because they believe that the other’s political party has acted in an immoral way, so for the opponent to still identify with that party brings their moral character into question. The worse the perceived harm caused by the other’s party, the more damning the other’s identification with the party should seem.

We proposed that theoretically, shared identity would precede group harm. Recognizing a target’s political affiliation should immediately produce a categorization of the self in relation to the target (shared identity). This should lead to assessments of the target’s group (group harm), which should then lead to moral judgments of the target—and finally, to empathy for the target’s misfortunes. If this theory is accurate, then any discrepancies between liberals and conservatives in ratings of shared identity and group harm would explain why liberals and conservatives differ in empathy for their opponents.

In summary, we first tested whether the intergroup empathy gap would differ according to participant politics. Second, we predicted that the size of the intergroup empathy gap would be associated with judgments of shared identity, group harm, and trait morality (in that order). Third, we predicted that people with higher perceptions of ingroup power would have smaller empathy biases, both within and between political groups (although this should initially be seen as smaller biases in shared identity, in line with our second prediction).

## Method

The design and procedure of Study 3 were essentially the same as in Studies 1 and 2, with an expanded range of measures and stimuli. Study 3 measured participant party affiliation (Republican vs. Democrat) rather than political ideology.

### Participants

Participants were recruited from CloudResearch and MTurk in September 2021. The final sample included 1,372 participants: 698 Democrats and 674 Republicans, *M*_age_ = 42.33, *SD* = 13.04; 734 female (54%), 630 male (46%), 8 gender diverse (< 1%); 1,030 White/Caucasian (75%), 92 Asian (7%), 88 Black/African American (6%), 57 Hispanic/Latinx (4%), 105 of other ethnicities (8%). Participants were each paid US$1.15.

### Design and Procedure

The survey used the same between-groups design as Studies 1 and 2. Participants read general information about the study and indicated their consent. Unlike in past studies, participants then answered key political items (party voting and ingroup power) to exclude participants who did not consistently vote Republican or Democrat. Participants then completed the experimental manipulation section, including mediators and outcome measures, followed by the manipulation check items, exploratory political items, and demographics.

### Stimuli

The stimuli in each condition were identical to those used in Study 1, with two exceptions. We varied the target’s occupation (randomly selected from five options). We also added two scenarios to the pool of empathy induction stimuli. The new occupations and empathy scenarios were drawn from previous research (Lucas & Kteily, 2018) to account for the perceived status of the target as a possible explanation for our previous results. See SOM (p. 5) for the complete scenarios.

### Measures

#### Predictor Variables

##### Participant Politics

Participants identified as either Republican or Democrat on their CloudResearch profiles.

##### Ingroup Power

This was measured using four novel items regarding the ingroup party’s ability to enact positive changes, for example: The Republicans/Democrats currently have the power to enact important policies, and the Republicans/Democrats currently have little ability to make any meaningful changes (reversed). Participants responded on a 7-point scale (−3 = *Strongly Disagree* to 3 = *Strongly Agree*). Items were averaged (α = .84).

#### Outcome Variables

In Study 3, we used only two measures of empathy: empathic concern and empathic intentions (α = .93 and *r* = .73, respectively), as these measures had shown the most consistent effects in Studies 1 and 2. Reducing the number of outcome measures also improved our power to detect effects.

#### Mediators

We included novel mediators in Study 3 and improved our measure of Target Morality:

##### Shared Identity

We used an adapted version of the “Inclusion of the Other in the Self” scale (Aron et al., 1992). Participants were prompted, “Please select the picture below that best describes the relationship between yourself and the person from the scenario.” Participants selected one of seven images showing increasingly overlapping circles labeled “Self” and “Other.” These images were coded from 1 (*no overlap*) to 7 (*strong overlap*).

##### Target Group Harm

Participants were asked, “How much do you agree or disagree that this person’s POLITICAL GROUP does each of the following”: for six novel items, such as *This group causes harm to me or people close to me* and *This group improves the wellbeing of the American people in general* (reversed). Participants responded on a 7-point scale (−3 = *Strongly Disagree* to 3 = *Strongly Agree*). Items were averaged (α = .97).

##### Target Morality

Participants were asked, “If you had to guess this person’s character, how much would you agree or disagree with the following statements?” (−3 = *Strongly Disagree* to 3 = *Strongly Agree*). Participants rated the two morality items from Studies 1 and 2, and the seven-item General Morality subscale from the Moral Character Questionnaire (Helzer et al., 2014), adapted to suit the context. Items included, *This person believes he or she acts in moral ways* and *This person does not usually do the right thing* (reversed). The nine items were averaged (α = .94).

## Results

For additional statistical detail (e.g., all pairwise comparisons), refer to SOM (p. 46).

### The Intergroup Empathy Bias

A 2 × 3 MANOVA on the two measures of empathy revealed main effects of relative group membership, *F*(4, 2,726) = 74.42, *p* < .001, Λ = 0.813, and participant politics, *F*(2, 1,363) = 6.815, *p* = .001, Λ = 0.990, but no two-way interaction, *F*(4, 2,726) = 2.25, *p* = .062, Λ = 0.993.

**Figure 5.**
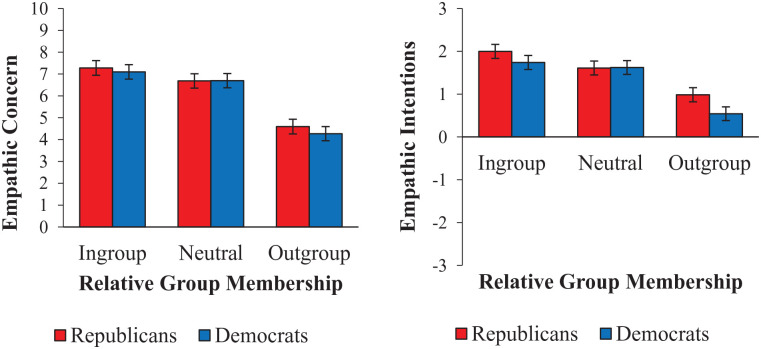
Mean Strength of Empathic Concern (Left) and Agreement With Empathic Intentions (Right) for Ingroup, Neutral or Outgroup Political Targets, as Rated by Republican and Democrat Participants. *Note.* Error bars indicate 95% confidence intervals.

We followed up these effects with univariate ANOVAs and pairwise comparisons, using a corrected threshold for significance of *p* = .034 for each test (Derringer, 2018) and Holm corrections for familywise error (Holm, 1979). Table 5 shows the univariate interaction terms and cell means, and Figure 5 shows the overall pattern of results.

**Table 5:** Cell Means and Omnibus Interaction Effects of Relative Group Membership and Participant Politics on Empathy Variables.

Variable	ω^2^	*df*	*df* error	*F*	*p*	Group condition	Means	
							Republicans	Democrats
Empathic concern	<0.01	2	1,366	0.51	.602	Ingroup	7.28	7.10
						Neutral	6.69	6.70
						Outgroup	4.60	4.27
Empathic intentions	<0.01	2	1,364	3.85	.021*	Ingroup	2.00_a_	1.74_a,b_
						Neutral	1.61_b_	1.62_b_
						Outgroup	0.98_c_	0.54_d_

*Note.* Differing subscript within a single row or column indicates where conditions differed, *p* < .034, after Holm corrections.

* *p* < .034.

Univariate ANOVAs revealed a main effect of relative group membership on empathic concern and intentions, with similar trends for both variables. Participants who read about an outgroup target felt less empathy than participants who read about either an ingroup or neutral target. Unlike in previous studies, participants who read about a neutral target felt less empathy than participants who read about an ingroup target.

There was a univariate main effect of participant politics on empathic intentions (but not empathic concern), showing that Republican participants had more empathic intentions than Democrat participants.

There was also a univariate Relative Group Membership × Participant Politics interaction for empathic intentions (again, not for empathic concern): all participants who read about outgroup targets showed less empathy than participants who read about ingroup or neutral targets, but Republican participants also showed more empathy to ingroup targets than neutral targets. Comparisons across participant politics showed that Republicans had more empathy than Democrats for outgroup targets.

### Moderated Mediation

We tested the proposed serial mediation using regression analyses (SOM, p. 65). These analyses showed modest indirect effects of relative group membership on empathy through shared identity, target group harm, and target trait morality, in that order (*B* = −0.11, *SE* = 0.02, 95% CI = [−0.15, −0.07]; *B* = −0.06, *SE* = 0.01, 95% CI = [−0.09, −0.04], for empathic concern and intentions, respectively). We found no moderation of this effect by either participant politics or ingroup power.

Given the weak mediated effect via shared identity and the lack of a moderated effect, we tested an alternative model that excluded shared identity. As in Studies 1 and 2, we tested for political asymmetries in moral judgment as an indirect explanation for political asymmetries in empathy. A two-way ANOVA showed no interaction between relative group membership and participant politics on the measure of target morality, *F*(2, 1,366) = 1.64, *p* = .195. However, a two-way ANOVA showed a significant interaction between relative group membership and participant politics on the measure of group harm, *F*(1, 903) = 9.67, *p* = .002. While both Republican and Democrat participants thought the opposing party caused more harm than the allied party, this effect was stronger for Democrats than for Republicans. Specifically, both parties felt equally confident that their ingroup did not cause harm (*M*_Dem_ = −1.88, *M*_Rep_ = −1.73), but Democrats more strongly agreed that Republicans caused harm (*M* = 1.67) than Republicans agreed that Democrats caused harm (*M* = 1.33).

**Figure 6.**
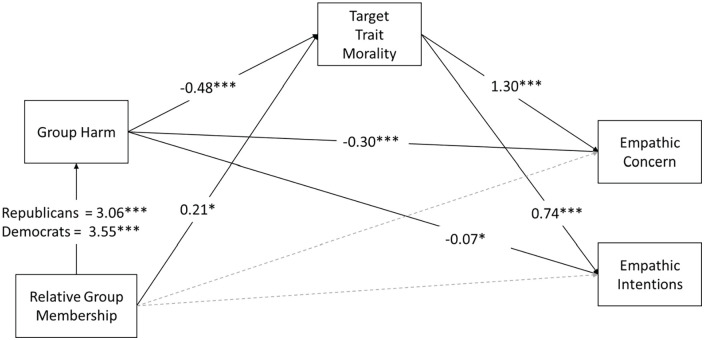
Path Diagram Showing Effects (Unstandardized) of Relative Group Membership on Empathic Concern and Empathic Intentions by Way of Perceptions of Group Harm and Target Morality, Moderated by Participant Politics. *Note*. Parallel mediation analyses were conducted on empathic concern and empathic intentions separately, but we have visualized these analyses as a single path diagram for comparison. Relative group membership was coded as outgroup = 1, ingroup = 0. Gray dashed lines indicate paths that were tested but were non-significant. *p < .05. ***p < .001.

We then tested moderated mediation models for the effect of relative group membership on empathic intentions and empathic concern, via group harm and moral judgment. The analysis models are visualized in Figure 6 and model coefficients are provided in Table 6. Analyses revealed that relative group membership was indirectly associated with both empathic concern and empathic intentions through group harm and perceived morality of the target, and these paths were moderated by the politics of the participant. Liberal participants who saw an outgroup target rated the target’s political group as more harmful, which was associated with lower ratings of the target’s morality, which was then associated with less empathy for the target. Conservative participants also showed these patterns but not as strongly as the liberal participants.

**Table 6.** Mediated Effects of Relative Group Membership on Empathic Concern and Empathic Intentions by Way of Perceptions of Group Harm and Target Morality, Moderated by Participant Politics.

Outcome	W (participant politics)	Indirect effect (ab)	95% confidence interval		Index of moderated mediation	95% confidence interval	
			Lower	Upper		Lower	Upper
Empathic concern	Democrat	−2.23	−2.631	−1.863	0.30	0.112	0.500
	Republican	−1.92	−2.304	−1.580			
Empathic intentions	Democrat	−1.27	−1.509	−1.058	0.17	0.061	0.284
	Republican	−1.10	−1.315	−0.903			

### Moderation by Ingroup Power

A series of regression analyses (detailed in SOM, p. 64) showed that perceived power did not moderate the effect of relative group membership on either shared identity, *F*(2, 1,370) = 0.16, *p* = .853, empathic concern, *F*(2, 1,372) = 0.23, *p* = .797, or empathic intentions, *F*(2, 1,370) = 2.65, *p* = .071. Those with higher perceived power did not differ to those with lower perceived power in the size of their outgroup biases. Nor did perceived power explain or mediate the effect of participant politics on empathy: Democrats perceived greater ingroup power than Republicans and ingroup power was positively associated with empathy, but Democrats showed less empathy than Republicans.

**Figure 7.**
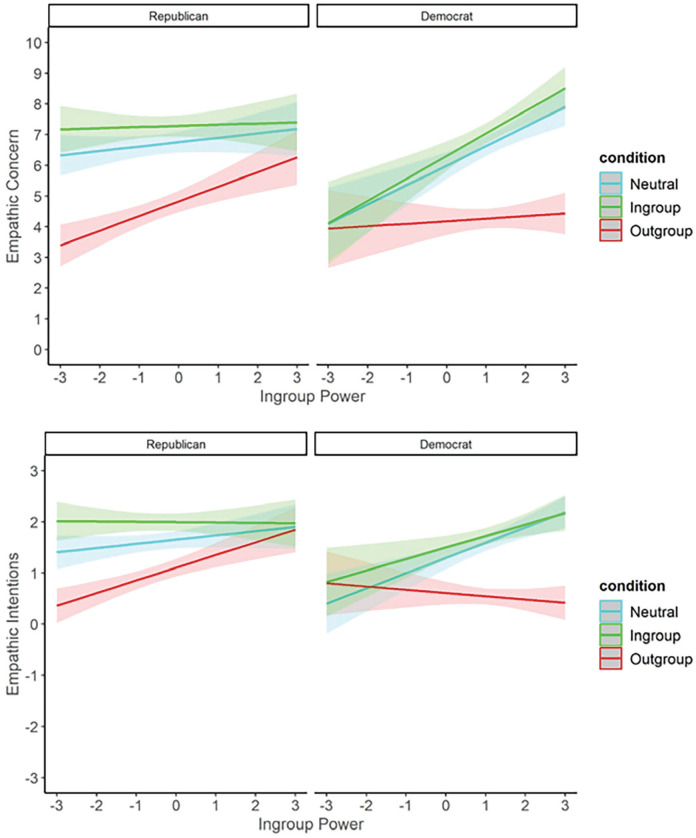
Mean Strength of Empathic Concern (Top) and Agreement With Empathic Intentions (Bottom) for Ingroup, Neutral or Outgroup Political Targets, as Rated by Republican (Left) and Democrat (Right) Participants, at Varying Levels of Perceived Ingroup Power. *Note.* Error bars indicate 95% confidence intervals.

As ingroup power did not explain the association between participant politics and empathy, we instead tested whether participant politics and ingroup power might interact to predict empathy. We ran three-way ANOVAs testing the effects of relative group membership, participant politics, and ingroup power on empathic concern and intentions. These revealed similar results for both outcome variables: main effects of relative group membership and participant politics (as described above), and also a main effect of ingroup power. These effects were qualified by three-way interactions for both empathic concern, *F*(2, 1,360) = 8.40, *p* <.001, and for empathic intentions, *F*(2, 1,358) = 9.97, *p* < .001. Follow-up simple effects of ingroup power at each level of relative group membership revealed different patterns for Republicans and Democrats, shown in Figure 7. Among Republicans, increased ingroup power was associated with increased empathic concern for outgroup targets, *t*(1,360) = 3.95, *p* < .001, *B* = 0.48, but not for ingroup targets, *t*(1,360) = 0.29, *p* = .773, *B* = 0.04, or neutral targets, *t*(1,360) = 1.21, *p* = .225, *B* = 0.14. Among Democrats, increased ingroup power was associated with increased empathic concern for ingroup targets, *t*(1,360) = 4.44, *p* < .001, *B* = 0.73, and also for neutral targets, *t*(1,360) = 4.47, *p* < .001, *B* = 0.64, but was not associated with concern for outgroup targets, *t*(1,360) = 0.54, *p* = .589, *B* = 0.08. The same patterns were found for the measure of empathic intentions (see SOM, p. 58). Thus, regardless of ingroup power, conservatives consistently reported high levels of empathy for the ingroup and neutral targets but varied their empathy for the outgroup, having more empathy for an outgroup member at higher levels of ingroup power. In contrast, liberals consistently reported low levels of empathy for the outgroup. However, they varied their empathy for the ingroup and neutral targets, having more empathy for these targets at higher levels of ingroup power.

## Discussion

In this study, we found mixed evidence of a political asymmetry in empathy. Democrats and Republicans showed similar empathic concern for their opponents. In contrast, Democrats had less empathic intentions for their opponents than Republicans did. However, this was a small effect that we did not have statistical power to detect reliably. Finally, Democrats had higher ratings of harm caused by Republicans, which was indirectly associated with larger biases on both empathic concern and intentions than those shown by Republicans. Where present, these political asymmetries followed the same patterns we found in Studies 1 and 2.

We replicated the finding that reductions in empathy for political opponents (relative to allies) are associated with harsher moral judgments of those targets, consistent with the claim that empathy is reduced because of moral judgment. We also elaborated on the origins of this moral judgment. To a small extent, reductions in empathy for an outgroup target were explained by an initial lack of shared identity with that target, which produced a chain of associations through increased perceptions of group harm, reduced target morality, and reduced empathy. However, the stronger explanation for reductions in empathy bypassed shared identity and began with perceptions of group harm. This process may explain the political asymmetries found in all three studies: if liberals (more than conservatives) perceived that the opposing political group was extremely harmful, that would lead them to see all political opponents as more immoral, and thus, liberals would have less empathy for their opponents than conservatives. We tested this suggestion again in Study 4.

Finally, we found limited evidence that perceived power explained the political asymmetry in empathy. Democrats perceived higher ingroup power and yet showed a larger empathy bias than Republicans, contrary to predictions. However, comparisons within each group showed conditional support for our prediction: higher-power Republicans behaved more generously to their opponents, as predicted, but Democrats’ empathy for their opponents was unaffected by ingroup power. If high-status groups minimize perceptions of conflict with low-status groups to maintain and legitimize status (Livingstone et al., 2021), it appears that only Republicans adopted this tactic. This could partly explain why conservatives were more empathic than liberals in our Studies 1 and 2 (under a conservative government). However, Democrats’ increased empathy for their *allies* in response to power in Study 3 is not consistent with the patterns seen in Studies 1 and 2 (at presumed low political power). As this finding was unexpected and without clear precedent, we tested whether it would replicate in Study 4.

## Study 4

The three previous studies have each found evidence of an asymmetry in political empathy. However, Studies 1 and 3 both lacked statistical power to detect this effect reliably. Therefore, to confirm that the political asymmetry exists and is statistically reliable in the United States, we repeated Study 3 with a much larger sample.

We re-tested our explanation for how these changes in empathy occur: that reductions in empathy for outgroup members occur because partisans call to mind the harm caused by the outgroup party; by association, any outgroup party supporter is perceived as more immoral, and people are reluctant to extend empathy to other people who are immoral. If this is the case, political asymmetries in empathy will arise when there are political asymmetries in group harm, through mutual association with moral judgment.

In Study 3, we found that Democrats and Republicans responded differently to changes in perceived ingroup power. In Study 4, we tested whether this result was reliable at higher levels of statistical power. Specifically, we tested whether, for Democrats, higher ingroup power would predict greater empathy for ingroup targets but not outgroup targets; but, for Republicans, ingroup power would predict greater empathy for outgroup targets but not ingroup targets. This study took place before and during the U.S. midterm elections, which allowed for a test of the hypotheses in a context where ingroup political power might be more variable within each group.

## Method

Study 4 followed the same general design and procedure as in Studies 1 to 3, but the survey was shortened to afford enough participants to reach statistical power.

### Participants

We recruited participants from Prolific in November 2022, stopping when the midterm election results were finalized on November 17. The final sample included 1,874 participants: 1,004 Democrats and 870 Republicans. *M*_age_ = 40.53, *SD* = 14.09; 984 female (53%), 869 male (46%), 21 gender diverse (1%); 1,501 White/Caucasian (80%), 138 Hispanic/Latinx (7%), 128 Asian (7%), 121 Black/African American (7%), with no other categories greater than 5% (in this case, ethnic categories were not mutually exclusive). Participants were each paid £0.60.

### Design and Procedure

The study used the same between-groups design as previous studies, except that we dropped the Neutral target condition to improve statistical power. We ordered the survey in the same way as in Studies 1 and 2: information and consent, followed by the experimental manipulation and responses, political items, demographics, and debriefing. This order was held constant (not counter-balanced) between participants.

### Stimuli

The stimuli were identical to those used in Study 3, except we held constant the target’s occupation (an assistant manager in retail, as in Studies 1 and 2).

### Measures

#### Predictor Variables

##### Participant Politics

Participants identified as either Republican or Democrat on their Prolific profiles.

##### Ingroup Power

This measure used the same four items and 7-point scale (−3 = *Strongly Disagree* to 3 = *Strongly Agree*) as in Study 3. Items were averaged (α = .80).

#### Outcome Variables

In Study 4, we only measured empathic concern (α = .95) as the most theoretically relevant measure of empathy. Reducing the number of outcome variables improved our power to detect effects.

#### Mediators

##### Target Group Harm

From the six items used in Study 3, we used the three items that measured perceived harm and excluded the reversed items that measured wellbeing (−3 = *Strongly Disagree* to 3 = *Strongly Agree*). Items were averaged (α = .97).

##### Target Morality

From the nine items used in Study 3, we used three of the items from the Moral Character Questionnaire (Helzer et al., 2014) (−3 = *Strongly Disagree* to 3 = *Strongly Agree*). Items were averaged (α = .87).

## Results

For additional statistical detail (e.g., all pairwise comparisons), refer to SOM (p. 82).

### The Intergroup Empathy Bias

We first ran a three-factor general linear model, including participant politics, relative group membership and perceived ingroup power as predictors of empathic concern. The model showed a strong effect of condition, η^2^_p_ = .25, *F*(1, 1866) = 504.85, *p* < .001, with some milder effects of politics, η^2^_p_ = .03, *F*(1, 1866) = 90.43, *p* < .001, and ingroup power, η^2^_p_ = .02, *F*(1, 1866) = 44.67, *p* < .001. These effects were qualified by two-way interactions, including a Condition × Politics interaction, η^2^_p_ = .02, *F*(1, 1866) = 33.41, *p* < .001, and ultimately by a three-way interaction, η^2^_p_ = .003, *F*(1, 1866) = 4.91, *p* = .027. We report this three-way interaction below, but first describe the two-way interaction (the baseline political asymmetry in empathy), in line with previous studies.

**Figure 8:**
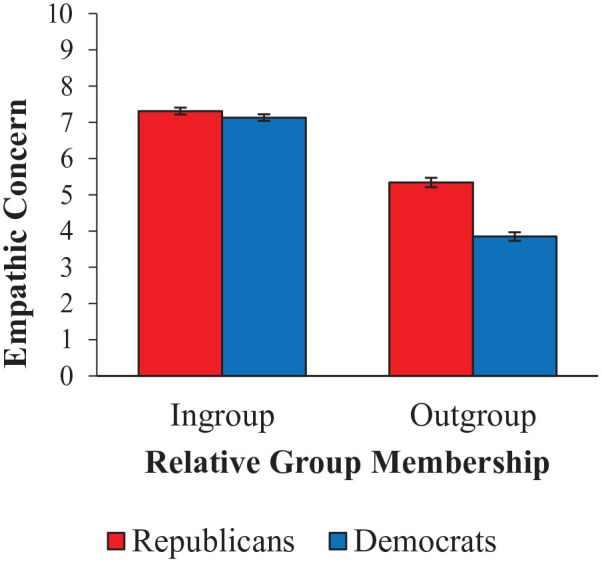
Mean Strength of Empathic Concern for Ingroup and Outgroup Political Targets, as Rated by Republican and Democrat Participants. *Note.* Error bars indicate standard errors of the mean.

Follow-up tests of the Condition × Politics interaction showed the same pattern of effects as in Studies 1 and 2: while both Democrats and Republicans showed less empathic concern for an outgroup target than an ingroup target, Democrats showed the stronger effect. As visualized in Figure 8, Democrats showed less empathy for their outgroup than Republicans did, and Republicans showed slightly more empathy for their ingroup than Democrats did.

### Moderation by Ingroup Power

**Figure 9:**
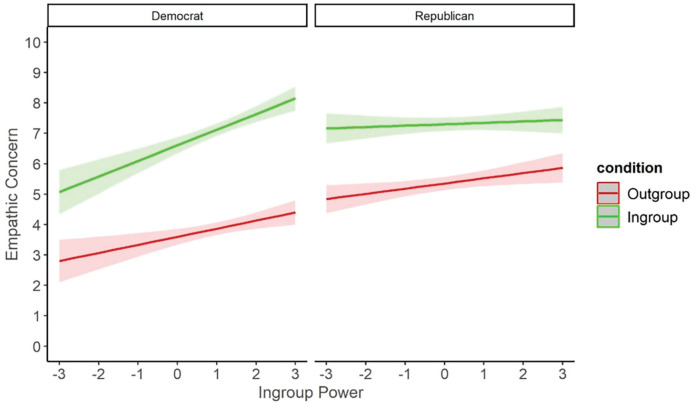
Mean Strength of Empathic Concern for Ingroup or Outgroup Political Targets, as Rated by Republican (Right) and Democrat (Left) Participants, at Varying Levels of Perceived Ingroup Power. *Note.* Error bars indicate 95% confidence intervals.

Follow-up tests of the three-way interaction showed the expected results for Republican participants: increased ingroup power predicted increased empathy for outgroup targets, *B* = 0.22, *t*(1,866) = 2.72, *p* = .007, but was not associated with empathy for ingroup targets, *B* = 0.06, *t*(1,866) = 0.71, *p* = .475. For Democrat participants, we found an attenuation rather than an extinction effect: increased ingroup power predicted increased empathy for ingroup targets, *B* = 0.55, *t*(1,866) = 5.85, *p* < .001, and also for outgroup targets, *B* = 0.33, *t*(1,866) = 3.58, *p* < .001. These results are visualized in Figure 9.

### Moderated Mediation

**Figure 10.**
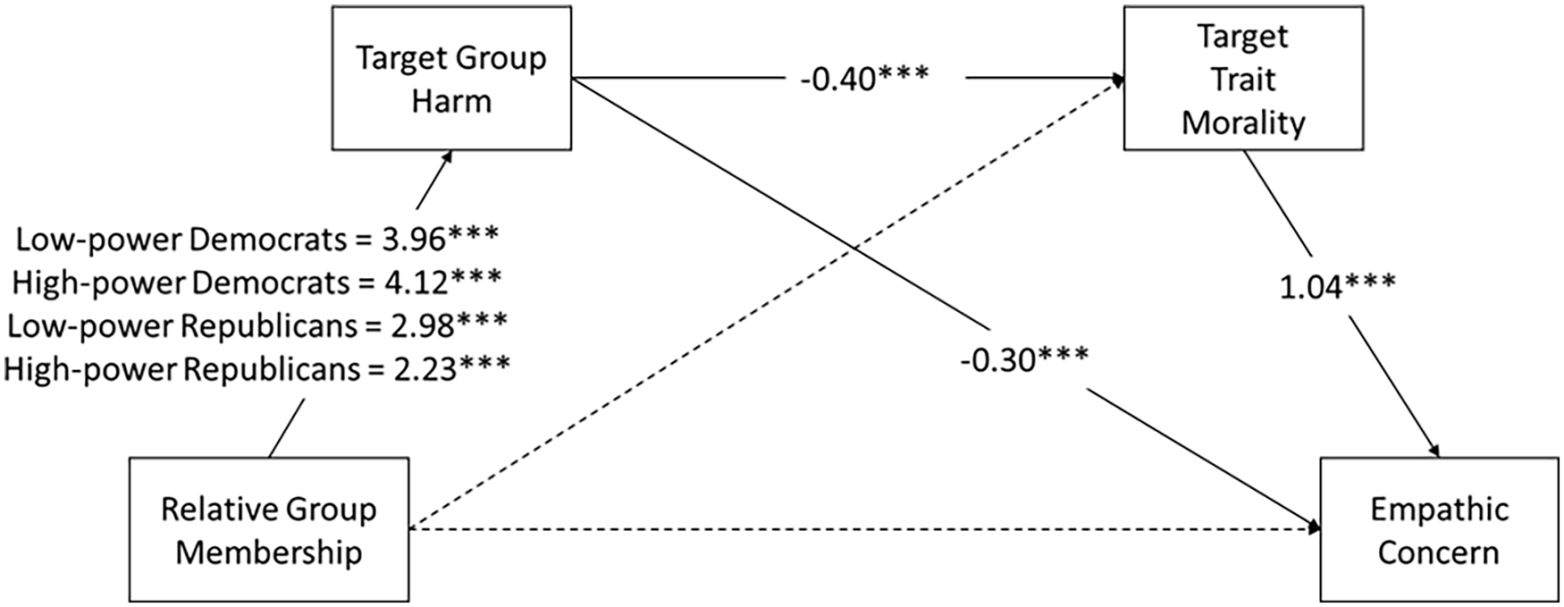
Path Diagram Showing Effects (Unstandardized) of Relative Group Membership on Empathic Concern by Way of Perceptions of Group Harm and Target Trait Morality, Moderated by Participant Politics and Ingroup Power. *Note*. Relative group membership was coded as outgroup = 1, ingroup = 0. Participant Politics was coded as Republican = 1, Democrat = 0. ***p < .001.

We used the PROCESS function in R to test a model where relative group membership, participant politics, and ingroup power interacted to predict empathic concern, via group harm and target morality. This model is visualized in Figure 10 and model coefficients shown in Table 7. We found evidence of moderated moderation by ingroup power, although the stronger effect was the moderation by participant politics (as in Studies 1–3). That is, seeing an outgroup target (relative to an ingroup target) had an indirect and negative effect on empathy by way of group harm and moral judgment. This indirect effect was slightly weaker among high-power Republicans and low-power Democrats, but even the “weak” effects for low-power Democrats were stronger than the “strong” effects for high-power Republicans.

**Table 7.** Mediated Effects of Relative Group Membership on Empathic Concern, Moderated by Participant Politics and Ingroup Power.

W (participant politics)	Z (ingroup power)	Indirect effect (ab)	95% confidence interval		Index of moderated mediation	95% confidence interval	
			Lower	Upper		Lower	Upper
Empathic concern							
Democrats	Low power	−1.659	−1.920	−1.423	0.153	0.071	0.240
	High power	−1.726	−1.984	−1.494			
Republicans	Low power	−1.250	−1.450	−1.072			
	High power	−0.936	−1.135	−0.753			

## Discussion

With a much larger participant pool in Study 4, we find a statistically reliable replication of the political asymmetry found in Studies 1 and 2 (and to some extent, Study 3): Democrats had larger empathy biases than Republicans and afforded less empathy to Republicans than Republicans did to Democrats.

We also replicate Study 3’s finding of the influence of ingroup political power. Democrats who perceived higher power had more empathy for other Democrats and did not increase their empathy for Republicans as much. By contrast, Republican participants who perceived higher party power had more empathy for Democrats but did not increase their empathy for their fellow Republicans as much (not at all). However, the effect size was very small, so we hesitate to make too much of this finding. Moreover, perceived political power did not appear to explain the political asymmetry in any straightforward way. For example, the *noblesse oblige* explanation—that people with high power will show more empathy to their opponents than people with low power—does match our results for both Republicans and Democrats independently but does not explain the discrepancy between them. Even the Democrats with the highest ratings of perceived power did not have more empathy for their opponents than the Republicans with the lowest ratings of perceived power.

In contrast, we again found evidence consistent with the argument that perceptions of group harm and moral judgment of the target can explain the political asymmetry. Democrats saw the Republican Party as much more harmful than Republicans saw the Democratic Party. This asymmetry in perceptions of group harm, through its association with moral judgments of specific targets, statistically explains the asymmetry in empathy.

## Meta-Analysis of Studies

To summarize effects across the four studies, we conducted a set of internal meta-analyses. First, we determined the aggregate effect of the empathy bias and the moderation by politics (the political asymmetry). We excluded neutral-target conditions and focused on the measure of empathic concern (the only measure included in all four studies). Following guidelines by Goh et al. (2016), we used a fixed-effects model to analyze the eight effects on empathic concern (four from conservatives and four from liberals) and weighted mean effects by sample size. Results are summarized in Table 8. By Cohen’s (1992) guidelines for effect sizes, there was a large overall empathy bias, *M*_r_ = .44, *Z* = 24.24, *p* < .001, such that people who saw ingroup targets showed more empathy than people who saw outgroup targets. The effect for liberal participants was large, *M*_r_ = .52, *Z* = 20.90, *p* < .001, and the effect for conservatives was medium, *M*_r_ = .36, *Z* = 13.38, *p* < .001. The liberal empathy bias was stronger than the conservative empathy bias, *Z* = 5.11, *p* < .001. Conservatives had more empathy than liberals for outgroup targets across studies, *M*_r_ = .21, *Z* = 8.08, *p* < .001, a small-to-medium effect.

**Table 8.** Meta-Analysis of Effects of Seeing an Outgroup Target (Relative to an Ingroup Target) on Empathic Concern.

	Liberals		Conservatives		Overall	
Study	*r*	*N*	*r*	*N*	r	*N*
Study 1	.39	178	.15	175		353
Study 2	.45	323	.22	302		625
Study 3	.48	464	.45	443		907
Study 4	.58	1,004	.39	870		1,874
Aggregate effects						
Mean *r*	.52	1,969	.36	1,790	.44	3759
*p*	<.001		<.001		<.001	

*Note.* Mean *r* = mean Pearson’s correlation, weighted by sample size.

We also tested the aggregate indirect effect of group membership on empathic concern via judgments of target morality—this represents the core mediation tested in all four studies. We specified fixed-effects models using the metafor package for R (Viechtbauer, 2010). Results, summarized in Table 9, showed a significant negative overall indirect effect of seeing an outgroup target on empathic concern via moral judgment, *aggregate IE* = -.53, *SE* = .02, *CI* [−.57, −.49]. This overall effect was moderated by participant politics, β = .26, *SE* = .04, *CI* [.18, .34]: the effect was smaller for conservative participants, *aggregate IE* = −.39, *SE* = .03, *CI* [−.44, −.33], than liberal participants, *aggregate IE* = −.65, *SE* = .03, *CI* [−.71, −.58].

**Table 9.** Meta-Analysis of Observed Effects of Seeing an Outgroup Target (Relative to an Ingroup Target) on Empathic Concern via Moral Judgment of that Target.

	Liberals		Conservatives		Overall	
Study	*IE*	*SE*	*IE*	*SE*	*IE*	*SE*
Study 1	−.48	.10	−.25	.08	−.39	.07
Study 2	−.47	.08	−.18	.05	−.33	.04
Study 3	−.73	.06	−.64	.07	−.69	.05
Study 4	−.70	.04	−.47	.05	−.60	.03
Aggregate effects						
*IE*	−.65		−.39		−.53	
95% *CI*	[−.71, −.58]		[−.44, −.33]		[−.57, −.49]	

*Note.* IE = standardized indirect effect; CI = confidence interval.

## General Discussion

In this article, we examined whether liberals and conservatives find it difficult to show empathy for a political opponent and if so, why. In doing so, we also explored differences between liberals and conservatives and when those differences show up. We confirmed a baseline intergroup empathy bias in all studies: when reading about someone in distress, people felt less empathy for political opponents than political allies or people of unknown political beliefs. As in past research into intergroup empathy biases (Cikara et al., 2014; Hasson et al., 2018), this bias was largely characterized by a reduction in empathy for outgroup targets relative to both neutral and ingroup targets and not by a boost in empathy for ingroup targets. The political empathy bias was present across multiple measures of empathy: political outgroup members were afforded less empathic concern and empathic intentions (as in the work of Hasson et al., 2018), as well as less perspective-taking and more empathic avoidance.

We also found a consistent asymmetry in this empathy bias. In each of our four studies (and in aggregate across studies) conservatives showed more empathy for liberals than liberals showed for conservatives. We found the same pattern in other judgments: of target likability, of similarity, and perceptions of group harm. This pattern—neither symmetrical (Brandt & Crawford, 2020) nor asymmetrical in the expected direction (Jost et al., 2020)—remains surprising. To explain the contrast between previous research and our own, we wondered if there had been a moderating influence of conservative, populist—perhaps even ‘Trumpian’—political power in our initial study. However, we found the same effect in both the United States and the United Kingdom, and under liberal and conservative governments and found only weak evidence that varying perceptions of political power could explain the presence or direction of this political asymmetry.

If conservatives indeed are more empathic than liberals—at least at the time of study—what explains these differences? We suggest that moral judgment partly explained both the general empathy bias and the political asymmetry in this bias. We consistently found associations between empathy for a target and judgments of the target’s morality and likeability (and, to a lesser extent, similarity to the perceiver). Accordingly, when someone encountered a suffering political opponent rather than an ally or neutral target, they had less empathy for them through associations with morality and likeability. This is consistent with the argument that it is difficult to empathize with political opponents because people feel that their opponents are immoral and unlikeable. Moral judgment was identified as a key barrier to empathy in all four studies in this article and explained additional, unique variance in empathy when accounting for the effects of liking and similarity. This is notable as moral judgment had not previously been shown to explain political empathy, though it is thought to facilitate political prejudice (Finkel et al., 2020).

In examining what creates such strong moralization of opponents, we found that the moral judgment of a target was associated with the perceived harm caused by that person’s group. Accordingly, when someone encountered a political opponent, they had less empathy for the target’s sufferings through shared associations with increased perceptions of harm and moral judgment. This is consistent with the explanation that moral judgment of one’s opponents (and thus low empathy) arises because we hold them responsible for the harms their group has caused.

These associations between moral judgment and empathy held true in general but differed in strength for liberals and conservatives; the associations were stronger among liberals. This suggests that at least some of the asymmetry in empathy arose from an asymmetry in moral judgment; liberals were more morally judgmental than conservatives, so that, this led to less empathy for conservatives. Furthermore, we found evidence of an asymmetry in perceptions of group harm in Studies 3 and 4 that explained the asymmetries in moral judgment. Democrats perceived that their opponents’ political party was more harmful, so they judged individual opponents to be more immoral than conservatives did (and thus afforded them less empathy).

Although this may describe the state of events in our research, why do we find these differences now, and not in past research? Even if we assume that personality differences between liberals and conservatives (Jost et al., 2003) are stable and reliable and influence empathic responses, it seems likely that group norms on each side might vary considerably in response to recent political events. These social pressures could overrule innate preferences. For example, in response to extreme events like the U.S. Capitol attack, conservatives may have sought to distance themselves from those who conducted the attack and encouraged norms of curiosity and tolerance for their opponents. At the same time, liberals may have responded by encouraging caution and intolerance of conservative transgressions. Even if this example is not correct, similar changes in group norms in response to other events would naturally lead to different patterns of results over time. This would explain why some more recent studies have found political asymmetries similar to ours (Conway et al., 2021; Eadeh & Chang, 2020; Sargent & Newman, 2021).

### Strengths and Limitations

Past research on political differences has been criticized for focusing on perceptions of targets more aligned with liberal than conservative values (Brandt & Crawford, 2020). We accounted for this by using explicitly liberal or conservative targets with the associated party affiliation. Where past work (Hasson et al., 2018) may have undermined the manipulation of target politics by presenting targets as political protestors (in conservative-leaning governments), we presented targets in various situations unrelated to their political identity in both liberal and conservative governments. As liberals and conservatives differ in their empathy for advantaged and disadvantaged targets (Lucas & Kteily, 2018), we varied and accounted for the perceived status of the target (see SOM, p. 88). Thus, we can be confident that our results accurately reflect judgments based on the target’s political identity (rather than attitudes toward protestors or status-based attitudes). We also measured and controlled for participants’ strength of identification with their group (SOM, p. 92), which allows us to be confident that our results are not due to ideological biases in our sample. Finally, research on partisan political relations tends to focus on U.S. samples. By including participants from the United Kingdom, we have demonstrated that the variability in the political empathy bias likely extends to other countries with two-party democratic systems.

We also acknowledge the limitations of these data. Although the studies here provide a “snapshot” of intergroup attitudes at each time point, we cannot draw conclusions about how individual people’s attitudes might have changed over time or at different levels of perceived power. Such information would be valuable if we want to create lasting change at a societal level or successful interventions at the individual level. It would also be useful to discover if these patterns of intergroup attitudes are present in other political systems—for example, in non-Western two-party democracies.

Finally, we acknowledge the limitations of our methods. We make frequent use of mediation techniques that are consistent with but cannot confirm causal relationships. If future work were to manipulate perceptions of outgroup harm or target morality and check for the effect on empathy, this would confirm that people have less empathy for their opponents (and liberals less empathy for conservatives) *because* of their perceptions of group harm. We also relied heavily on self-report, attitudinal data collected through online populations. Although the use of self-report attitudinal data is widespread in this and other domains, behavioral measures of empathy would help resolve some of the tension between what people say (conservatives report higher empathy for liberals) and what people do (e.g., hate crimes are overwhelmingly committed against left-leaning targets; see Badaan & Jost, 2020). Finally, it is possible that the online populations we recruited from are not representative of offline populations. For example, conservatives who participated in our studies may be more moderate than conservatives who did not participate.

### Implications

Our research suggests that conservatives have more favorable opinions of liberals than liberals have of conservatives and thus conservatives extend more empathy to liberals in times of need. What we should do with this information is a matter of interpretation. On one hand, if liberals will not extend empathy to conservatives who are suffering, it seems that the problem lies with liberals, that they are being overly critical of their opponents and failing to engage in prosocial behaviors that their opponents would extend to them. On the other hand, we have argued that much of the difference in empathy is because of differences in perceptions of harm caused by the opponent’s political group. The fact that these are unequal *perceptions* of harm does not mean they are unfounded or without reason, in the same way that a victimized group may have good reason to be upset at another group that has harmed them. It does not necessarily follow that the victimized group should no longer feel hurt because the perpetrator group is sometimes sympathetic and benevolent. To build trust between groups in this situation, it may be necessary for conservatives to acknowledge and repair the harms they have caused, as well as for liberals to give individual conservatives the benefit of the doubt, considering conservatives hold relatively positive attitudes toward liberals.

More broadly, the existence of empathy biases across political lines points to great difficulty in addressing the problems of affective political polarization even when liberals are no more biased than conservatives. If empathy for suffering others is reduced simply because they belong to an opposing political group, it will be challenging to promote goodwill and respect toward political opponents who are *not* suffering. Worse, direct interventions to improve empathy may backfire. Our research suggests that direct approaches may fail to acknowledge the underlying reasons for low empathy: partisans see their political opponents (immoral people who support harmful groups, who are unlikeable and “other”) as being so fundamentally in opposition to themselves that empathy is unrealistic and undesirable. As one participant commented, “It’s like trying to remain civil to someone who just stabbed you.” Indirect approaches that target these underlying reasons may be more effective in improving empathy. For example, increasing perceptions of variability among the opposing party’s supporters reduces the size of the intergroup empathy bias (Cikara et al., 2014); this approach may work because it weakens the association between group harm and moral judgment of the individual.

Overall, this research suggests that moral judgment maintains a lack of empathy between political opponents. To improve empathy across political divides, we can take steps to address moral judgments on both sides of politics. However, it is also worth acknowledging that liberals and conservatives can differ in the extremity of these judgments—and not always in the direction we might expect.
